# Patterns and implications of plastic accumulation in mangrove ecosystems and sandy beaches in Western and Central regions of Ghana, West Africa

**DOI:** 10.1007/s11356-025-36359-7

**Published:** 2025-04-22

**Authors:** Geslaine Rafaela Lemos Gonçalves, Curtis Grey, Albert Koomson, Joseph Aggrey-Fynn, Benjamin Kofi Nyarko, Bhavani Emma Narayanaswamy

**Affiliations:** 1 University of the Highlands and Islands, UHI House, Old Perth Road, Inverness, IV2 3JH UK; 2 Scottish Association for Marine Science, Oban, Argyll, Scotland PA37 1QA UK; 3 Department of Fisheries and Aquatic Sciences, University of Cape Coast, Cape Coast, Ghana; 4 Department of Geography and Regional Planning, University of Cape Coast, Cape Coast, Ghana

**Keywords:** Plastic litter, Water sachets, Plastic bag, Polyethylene, Single-use plastics, Waste management

## Abstract

Plastic pollution has become increasingly apparent in sandy beach zones and aquatic environments, creating more than just visual pollution. Impacts are observed in many environmental and social levels, including the fishing communities that depend on the coastal environment for their livelihoods. Plastic pollution was assessed on the sandy beaches and mangroves of Ghana’s Western and Central regions. The study’s objective was to determine the composition, abundance and sources of plastic litter at four different sites during the wet and dry seasons. Samples were collected from within 50 cm^2^ quadrats placed randomly along four transects at each site. Plastic litter was classified according to the OSPAR guide. A total of 1895 plastic litter items with a combined weight of ~ 3000 g, representing 30 plastic categories, were collected. The average number and weight of plastic litter items were 19.73 ± 31.37 number of plastics per 50 cm^2^ and 32.59 ± 45.47 g per 50 cm^2^. The minimum and maximum plastic litter items were 0 to 159/50 cm^2^. The highest amount of plastic litter was found in Ghana’s Central region. The total plastic litter weight is the variable that present statistical difference between the wet and dry season period. Bags, bottles, and fragments were the most common plastic items found, with 70% of the plastic litter being land-based, with the most frequent polymer types found in this study being polyethylene (~ 54%) and polypropylene (~ 20%). Mangrove regions act as sinks, specifically trapping plastic bags, contrary to the sandy beach areas, which mainly comprise plastic bottles that accumulate in these regions. Regions that have numerous communities, or are urbanised centres, tend to have higher levels or plastic litter. West Africa generally has poor waste management, absence of safe drinking water, and high levels of single-use plastics which are some of the main reasons for increased levels of plastic litter specifically in Ghana. Urgent actions are needed to prevent, mitigate and control plastic pollution in Ghana and the wider region.

**Graphical abstract:**

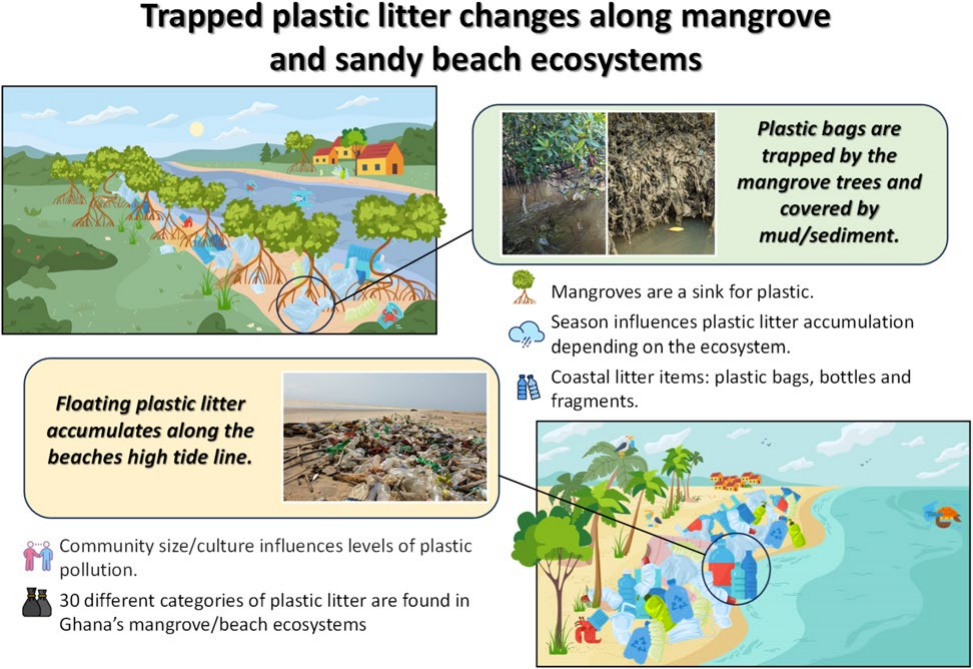

**Supplementary Information:**

The online version contains supplementary material available at 10.1007/s11356-025-36359-7.

## Introduction

Low-cost, flexible, lightweight and durable plastics have many uses in our daily lives (Jeyasanta et al. 2020; Zahari et al. 2022). Single-use plastics lead to the rapid accumulation of plastic waste in the environment and invariably enter the ocean via numerous pathways (Zahari et al. 2022). Plastic has become the most abundant solid contaminant in the marine environment (Galgani et al. 2015), including in sandy beach areas. Even with the efforts carried out under the Convention for the Protection of the Marine Environment of the North-East Atlantic managed by the OSPAR Commission to reduce marine litter (especially plastic), into levels that do not cause harm to the coastal and marine environment (Bergesen et al. 1998; Wenneker and Oosterbaan 2010), unfortunately this is far from being achieved. Borrelle et al. (2020) estimated that 23 million metric tons of plastic entered the oceans in 2016, with the global economic cost of marine plastic pollution being ~ US$500–2.5 billion yr^−1^ (Beaumont et al. 2019).

The amount of plastics entering the water bodies and the ocean is more pronounced in many Lower Middle-Income Countries (LMIC), mostly because of inefficient waste management systems (Acquah et al. 2021) and inadequate recycling infrastructure, resulting in improper waste disposal in the field or directly into water bodies, hence accumulation of plastic waste in vulnerable ecosystems. West Africa is composed of 16 countries, six of which are LMICs facing significant challenges in managing plastic waste (Akindele and Alimba 2021).

Ghana is one of the LMIC countries most affected by waste generation and has been ranked the seventh most polluted country in the world by the WHO/UNICEF Joint Monitoring Report (Smith-Asante 2015). This is due to inappropriate waste collection and treatment in virtue of financial challenges, population expansion, absence of safe drinking water and others (Awuah 2018). Single-use plastics are the main generator annually in Ghana (Addo et al. 2022). Ghana generates around 0.84 million tonnes of municipal plastic waste each year—a total that is growing annually by 5.4%, an increase in per capita plastic consumption of 3.4% per annum (Ghana National Plastic Action Partnership (NPAP), 2021). Ghana has an indiscriminate use of plastic products with no or little legislation (if any) to regulate its use (Adu-Boahen et al. 2022), just 10% of solid waste is properly disposed of in Ghana (Miezah et al. 2015). If the plastic litter generation continues in Ghana, it is expected that the plastic litter entering the water bodies will rise by 190% to 228,000 tonnes per year by 2040 (based on 78,000 tonnes per year in 2020) (Ghana National Plastic Action Partnership (NPAP), 2021). Plastic pollution impacts the Ghanaian artisanal fishery, with it accounting for ~ 32% of the total catch and ~ 42% by weight of the fish landed (Gbogbo et al. 2023), affecting the food and financial income of the local Ghanaian coastal communities that depend on the coastal beach environments to survive (Ghana National Plastic Action Partnership (NPAP), 2021; Gbogbo et al. 2023). Over the years, plastic has generated public health issues in Ghana, blocked drains and/or being carried by runoff water entering lakes, rivers, coastal areas, mangroves, and the sea (Galgani et al. 2000). Meijer et al. (2021) estimated that over 1000 rivers accounted for 80% of global annual plastic emissions into the sea, ranging between 0.8 million and 2.7 million metric tons per year. Ghana’s coastal region is 600 km long, of which almost half is sandy beaches (Dei 2010), with a mangrove area of ~ 137 km^2^ (United Nations Environment Programme, UNEP 2007). Mostly of the sandy beaches in Ghana suffer from coastal sand mining activities (Mensah and Mattah 2023) and plastic pollution. For example, on Accra’s sandy beaches up to eight plastic litter items can be deposited per m^2^ during each tidal cycle (Gbogbo et al. 2023).

Many ecological services are provided by coastal regions, such as coastal protection, water collection and purification, wildlife preservation and diversity, carbon sequestration, climate change mitigation and recreational/educational tourism (Drius et al. 2016) and, as a result, are an essential ecosystem, both for humans and wildlife. Sandy beaches provide a wide range of ecosystem services, such as sediment storage and transport, wave dissipation, i.e., coastal protection, breakdown of organic materials and pollutants, water filtration and purification (Narayan et al. 2017), nursery areas for juvenile fishes, nesting sites for a variety of organisms, human recreation and food sources (Defeo et al. 2009). Mangroves act as nursery grounds that support > 30% of commercial fish and other marine species in tropical and sub-tropical regions (Yamazaki et al. 2014), and contribute to food security by the provision of other food sources for humans, e.g., fruit, honey, etc. Mangroves are extremely important as carbon (C) sinks, with ~ 15% of the global C sequestration occurring in mangroves (Alongi 2014), largely due to the structure of the mangrove trees (prop roots) and leaf litter production.

Coastal areas as mangroves becoming more greatly impacted by plastic litter (Morales-Caselles et al. 2021; Smith and Turrell 2021; Allison et al. 2023), especially when they are close to cities, riverine systems, industrial areas, fishing communities, aquaculture and agricultural farms, and tourist hotspots (Kaandorp et al., 2021). Mangrove cover in Ghana has declined over the years due to, e.g., overexploitation, construction and pollution, with only 1.5% of mangroves occurring in protected areas (United Nations Environment Programme, UNEP 2007). Nunoo and Agyekumhene (2022) report that mangroves in the Central region of Ghana are heavily polluted by plastics that have been trapped by the tree roots. Recently, higher levels of microplastic have been reported for mangrove regions in Central and Western region of Ghana by Faseyi et al. (2022) and Gonçalves et al. (2025). In general, there is an overall deficit in knowledge and insufficient data regarding plastic pollution in Ghanaian environments such as mangroves and coastal environment in general (Adu-Boahen et al. 2022; Acquah et al. 2021). The consequences of plastic litter on Ghana’s beaches and mangroves are far-reaching and devastating. Research has shown large quantities of plastics along Ghana’s coastal environments that affect the ecosystems and the organisms, as well as negatively impacting finances generated from fishing, recreation and tourism (Ghana National Plastic Action Partnership (NPAP) 2021; Adu-Boahen 2024).

Plastic pollution accumulation is directly influenced by the environment characteristics that can influence plastic litter abundance, distribution, sources and composition, as well as physical processes such as currents, winds and waves which can transport plastics differently in each region (van Sebille et al. 2020; Galgani et al. 2015; Buhl-Mortensen et al. 2022). Plastic becomes a persistent pollutant when buried (Gündogdu and Çevik, 2019), e.g., in mangroves and sandy beaches. The morphology of mangrove trees and the influence of tides on river borders result in increased retention and accumulation of plastic (Govender et al. 2020; Maghsodian et al. 2022), leading to trapping of all sizes of plastic, with some studies finding that > 50% of some mangrove forest floors are covered in plastic debris (Martin et al. 2019; 2020; Walther and Bergmann. 2022). Plastic litter is one of the main items responsible for mangrove degradation, negatively affecting tree density, the growth of new tree seedlings and reducing mangrove health quality (Suyadi and Manullang 2020). They are mostly retained by the seedlings, roots and branches of the mangrove trees, which physically break down seedlings and prevent photosynthesis and root respiration (Suyadi and Manullang 2020). Despite their durability, plastics entering the environment break down over time into varied-sized fragments, such as macroplastics (≥ 25 mm), mesoplastics (5–25 mm) and microplastics (≤ 5 mm) (Barnes et al. 2009; Andrady 2011; Suyadi and Manullang 2020). Microplastics can be classified as primary MPs when manufactured to be < 5 mm in size, or secondary MPs from the result of breakup/fragmentation of larger plastic items via different process as biological, physical and chemical (Rochman et al. 2019).

According to Nelms et al. (2022), to understand the spatio-temporal abundance, distribution and composition of plastic litter datasets with broad geographical coverage are needed to implement strategies targeted at mitigating the problem. However, this can be costly, time-consuming and labour-intensive (Nelms et al. 2022). Nonetheless, beach-clean monitoring campaigns based on the OSPAR guide (Wenneker and Oosterbaan 2010) provide a useful tool for understanding the composition, distribution and abundance of plastic litter (Nelms et al. 2017; Smith and Turrell 2021; Allison et al. 2022), and at a low financial cost. However, unlike coastal beaches, mangroves do not have a litter monitoring protocol or programme like that for sandy beaches. Studies and tools estimating plastic litter in mangrove areas are scarce; the few studies that exist normally use the transect/quadrat methodology to quantify the surface plastic litter in these ecosystems (de Almeida Duarte et al. 2023; Cappa et al. 2023; De et al. 2023). The low amount of research in these areas could be related to the difficulties in accessibility to the sites and collecting the litter; additionally, the visualisation of plastic litter in mangroves is camouflaged/hidden by the organic matter (Kesavan et al. 2021). Lack of standardised sampling methodologies, litter classifications and sampling efforts are the major constraints to studies undertaken in mangrove environments. This study proposes to estimate the quantity of plastic litter in Ghana’s coastal regions at four coastal sandy beaches and four mangrove sites in the Western and Central regions of Ghana. Thus, we classify the plastic litter found in these sites to understand the plastic pollutions levels and sources. We expect that the regions more preserved in the Western region with low population would present low levels of plastic pollution. During the study, periods of wet and dry season are sampled to investigating how rain and dry periods can influence plastic litter distribution and accumulation in mangrove and sandy beaches between sites and regions. This research increases our knowledge about areas with higher plastic accumulation levels as well as the sources of these litter items and the ecosystem/time frame with higher accumulation. This provides important data and information which can be used to help conserve Ghanaian coastal ecosystems and mitigate plastic pollution impact in these areas.

## Material and methods

### Study locations

**Fig. 1:**
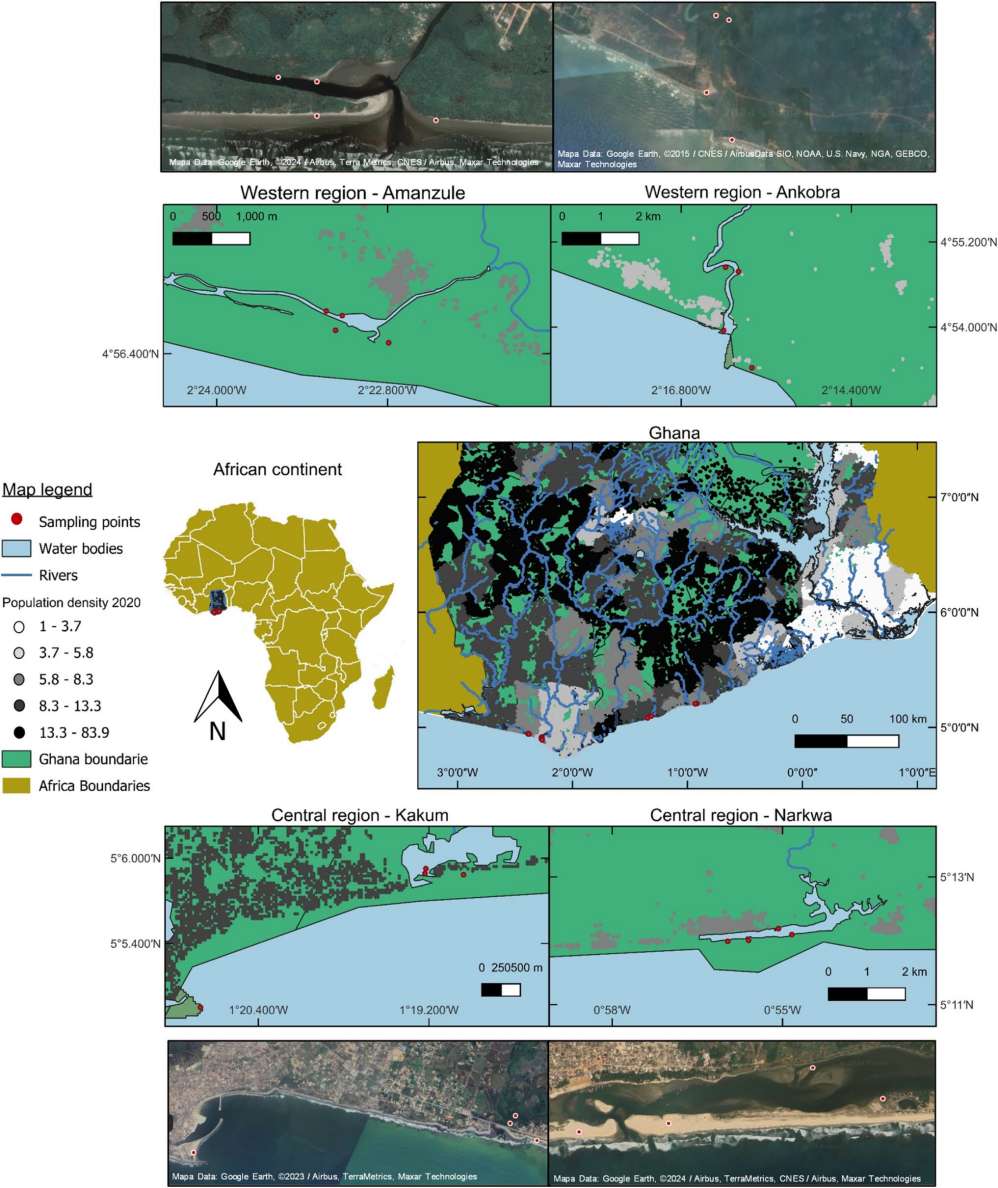
Sandy beaches and mangrove areas sampled (red dots) for plastic litter in Ghana, Africa. At the top of the figure are the two regions representing the Western region of Ghana (Amanzule and Ankobra), and at the bottom are the central areas studied (Kakum and Narkwa). The graded colour represented Ghana’s population density for 2020 (source: Ghana: High-Resolution Population Density Maps + Demographic Estimates). The same areas sampled are also represented by Google Earth satellite images to signify the actual shape of the areas

Samples were collected at four different coastal mangrove and sandy beach regions in the Western (more preserved regions—Amanzule and Ankobra) and Central (Kakum and Narkwa with more anthopogenic pressure) regions of Ghana, as presented in Fig. 1—the population density (2020) can be visualised in the research areas as well as the actual status of the regions according to satellite images.

The coastal beaches in the study, like most beaches in the Gulf of Guinea, appear to be dissipative in nature (Bosboom and Stive 2023). Dissipative beaches are characterised by gentle slopes, fine sediments and high wave energy, which can influence plastic litter deposition patterns (Bosboom and Stive 2023). The studied regions were selected according to the beach’s similarity and the level of anthropogenic pressure. The regions included sandy beaches with one estuary that was lined with relatively intact, healthy mangroves yet exploited by the local fishing community (Amanzule); two estuaries had degraded mangroves—one with clear-felled patches and beaches exploited by resorts and fishery (Ankobra), and the other mangrove that is subject to long-term selective deforestation with beaches heavily exploited by fishery and tourism (Kakum); the fourth estuary was lined with mud and sand flats, with almost no mangroves within a fishery community near the beach (Narkwa) (Hall et al., 2024, Gonçalves et al. 2025).

The two sites in the Western region, Amanzule and Ankobra, are characterised by flat, wide beaches and are predominantly shaped by low-energy wave events (Angnuureng et al. 2022; 2023). These areas are situated in the Ellembelle district which has a low population density (∼121 people per km^2^) (Ghana Statistical Service 2021). These regions are better preserved and less populated than Kakum and Narkwa in the Central region. The Western region falls within the wet equatorial climate zone, characterised by two distinct wet seasons occurring from May to June and October to November (Ajonina et al. 2014). The Amanzule region (~ 4°56′41.6″N and 2°23′12.4″W) is recognised as a preserved area featuring mature forest cover and low anthropogenic pressures, with coastal areas exploited for artisanal fishing (Faseyi et al. 2022). Despite lacking formal conservation status, the Amanzule forest has been traditionally protected and revered as the “dwelling place of the Gods” (Adupong et al. 2013; Ajonina et al. 2014). The other site studied is Ankobra (~ 4°54′51.3″N and 2°16′10.3′W); the Ankobra river and its associated estuarine ecosystem play crucial roles as sources of fish and livelihood for numerous communities especially the Asanta community that live in the region surrounding the estuary (Effah et al. 2021; Osman et al. 2016). However, this area is facing a rapidly increasing risk from pollution (MoFAD - Ghana Ministry of Fisheries and Aquaculture Development and Fisheries Commission 2020). The coastal area in this site is also exploited by artisanal fishers and for tourism (resorts nearby need to clean the waste from the beach on a daily basis to make it more attractive to tourists (personal observation)).

The two sites, Narkwa and Kakum, in the Central region are in the dry equatorial zone, characterised by wet season peaks from May to June and September to October (Adotey et al. 2022). Narkwa (~ 5°12′23.4″N and 0°55′03.7″W) has a wide coastal estuary featuring areas of sand flats and mudflats along with sparse mangrove cover near the mouth. The population density in this region is 210 people per km^2^ (Ghana Statistical Service 2021), and this area has a community which depends on artisanal fishing. Other human activities in this region, include construction and deforestation between the estuary and the sandy beach as well as pollution from domestic waste, resulting in compromised water quality (Takyi et al. 2022). The second site, Kakum, (~ 5°05′55.6″N and 1°19′13.2″W), is situated along the Cape Coast – Takoradi trunk road between Elmina and Cape Coast; the Kakum and Sorowie rivers flow through this region, which has undergone rapid urbanisation and industrialisation within the Central region. These rivers form an integral part of the estuary and have been subject to long-term resource exploitation, resulting in patchy and stunted mangrove forests, the communities also use the mangrove environment to dispose of solid and liquid waste (Fianko et al. 2007). The Cape Coast Metropolitan district is characterised by a high population density of 1557 people per km^2^ (Ghana Statistical Service 2021). The coastal sandy beach in Kakum is parallel to the main road with a heavy flux of vehicles; this area has more tourist sites with bars and restaurants on the beach, with a few fishery activities. Access to the sandy beach areas near the estuary is limited by the construction of a sea defence wall (Mensah 2022).

All coastal areas (sandy beaches and mangroves) studied in Ghana’s Western and Central regions had higher quantities of waste, such as plastic litter accumulating along the high tide line of the beaches (Mensah 2022). Two sites of the sandy beaches of the regions used as sample points presented to have a beach cleaning effort for tourist reasons: Ankobra near the resort and Kakum at the Elmina castle direction.

### Sampling collection

**Fig. 2:**
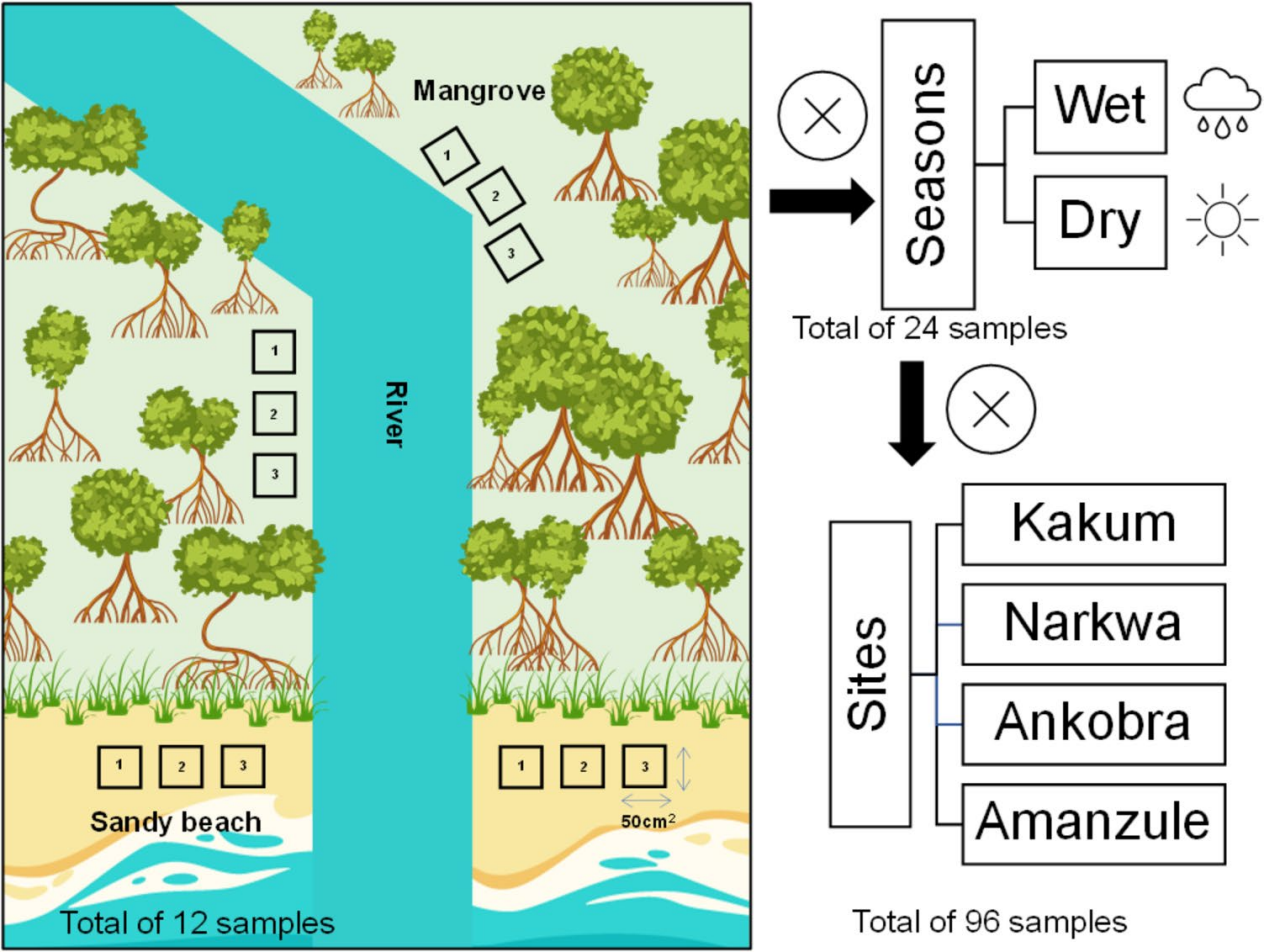
Representation of sampling collection done at each site studied. In the mangroves two transects (at straight and curve points of the mangrove river) were selected, where three quadrants replicate of 50 cm^2^ were sampled in each transect—with a total of six quadrants. At the sandy beach sites two transects were selected one in each side of the river (right and left) when it connects to the sea; in each transect three quadrants replicate of 50 cm^2^ were sampled—total of six quadrants. Thus, each site sampled had a total of 12 samples, which were collected during wet and dry season period—becoming 24 samples per site studied. Finally, we had four sites that were sampled totalling 96 quadrants samples (24 samples per site multiplied by four sites)

The surveys were conducted during two distinct periods of the year: October 2022 (150.77 mm of precipitation, temperature: min 22.5 °C – max 31.7 °C) during the wet season and in January 2023 (9.52 mm of rainfall, temperature: min 20.6 °C – max 34.0 °C) during the dry season (World Bank 2021). Samples were collected along two transects in both the coastal mangrove and sandy beach regions at each site (Kakum, Narkwa, Ankobra and Amanzule). At each site four transects were sampled, with three quadrat collection points, resulting in a total of 12 samples, six in the mangroves and six in the sandy beaches. Each site was visited once during the wet and dry season. In total there were 96 quadrat sampling points spread over the four sites and two seasons (Fig. 2). Along each transect, the samples were collected at the high tide line in both the mangrove and sandy beach areas.

The sandy beach transects were sampled on both sides of the beach where the river connects to the sea, on the right- and left-hand sides. At Kakum, it was not possible to do this for one side because of the sea defences; as result we relocated our sampling collection to the closest beach, Elmina Castle. Samples were collected at the high tide line at the mangrove and sandy beach region. Samples were collected using a quadrat of 50 cm^2^ (50 × 50 cm in size) (adapted from Suyadi and Manullang 2020). The plastic litter at the sediment surface (top 2.5 cm) inside each quadrat was collected using a stainless-steel trowel.

### Sampling processing and analyses

The plastic litter at the top of the sediment inside the quadrats were collected and stored in sampling bags and labelled. Sequentially, the top 2.5 cm sediment samples were washed over a 4-mm mesh sieve, and the plastic was separated and stored in plastic bags (Suyadi and Manullang 2020). These plastic items (> 0.5 cm) were rewashed at the laboratory to remove any adhering material (e.g., sand and organic matter). The samples were placed in trays covered with paper towels and left to air-dry for 15 days. All items were classified into litter categories defined by OSPAR (OSPAR Agreement 2020–01). For this study, only litter items in plastic categories were used (*n* = 77). A full list of litter item categories and corresponding OSPAR ID values are provided in Supplementary material (Table S2). For each plastic litter category, a count of litter items and dry weight (g) were recorded (Allison et al. 2023). Dry weight was measured using Brecknell and Secura weighing scales (capacity: 3 kg–0.1 g; 510 to 0.001 g resolution, respectively).

The average beached loading of plastic litter items per 50 cm^2^ was calculated, both by count – number of items (NP/50 cm^2^) and weight (g/50 cm^2^), for assessment of the variability of beached loadings between mangrove and sandy beach regions as well between the survey sites: Western and Central regions of Ghana (adapted from Allison et al 2023). To understand the origins of the plastic litter, the composition of each area sample was analysed by dividing all individual plastic litter item categories (*n* = 77) into three groups based on their proposed source: i.e., land-sourced, marine-sourced or of unknown source (Table S2), based on a survey protocol from the Marine Conservation Society (MCS “Where does this come from?”) and OSPAR guidelines (OSPAR Commission 2020; Allison et al. 2023) where the sources of litter are categorised according to their use before becoming litter, and also to ensure consistency with other monitoring studies.

### Plastic identification and characterisation

An Attenuated Total Reflection-Fourier Transform Infra-Red spectrometer (ATR-FTIR Thermo Scientific Nicolet iN10 and iZ10) was used to identify the polymer types of the most abundant categories of plastic litter using the FTIR in reflection and µATR collection mode (wavelength ranging from 4000 to 600 per cm^−1^). Each final spectrum is the product of 16 coded spectra with a spectral resolution of 8 cm^−1^. Spectra were compared with six inbuilt polymer libraries (Thermo Fisher) to aid identification and processed through atmospheric suppression and baseline corrected when necessary.

### Data processing and statistical analyses

Shapiro–Wilk test was performed to check for normality, followed by Levene’s Test to test the homogeneity of our data. As the data were found not to be parametric multiple comparisons were analysed using the nonparametric Kruskal–Wallis statistical test. If the test indicated significant differences, a post-hoc pairwise Wilcoxon test was conducted. The level of significance (5%) was set at *p* value of 0.05. All statistical analyses were performed using R ver. 4.4.2 (R Core Team (2024).

## Results

### Plastic litter abundance

A total of 48 m^2^ was surveyed across the four regions at the mangrove and sandy beach regions during wet and dry seasons, with a total number of 1895 plastic litter items that altogether weighed 3128.64 g, giving an average weight of 1.5 g per plastic litter item sampled. The average number of plastics items for all the study sites and location was 19.73 ± 31.37 NP/50 cm^2^ (around 79 items per m^2^) with a plastic weight of 32.59 ± 45.47 g/50 cm^2^ (~ 130 g per m^2^). The minimum and maximum number of plastic litter items found was 0–117/50 cm^2^ on the sandy beaches and 0–159/50 cm^2^ in the mangrove regions (Table S1).

To quantify the plastic litter abundance relative to the survey region (mangrove + sandy beach) the data were separated between the Western and Central regions. Before the analyses, a statistical test was performed between the sampling points at the sites Kakum and Ankobra, to check if the beach cleaning performed in these areas influences the abundance and weight of the plastic litter in these sites. However, statistical tests did not present any differences (Kruskal–Wallis test, *χ*2 < 1.35, df = 1, *p* > 0.05, see full result in Table S2). The average number and weight of plastic items recovered from the mangroves in the Western region were 2.29 ± 3.04 NP/50 cm^2^ and 10.63 ± 30.41 g/50 cm^2^ respectively, whilst the sandy beaches had 7.20 ± 7.23 NP/50 cm^2^ and 30.53 ± 44 g/50 cm^2^ respectively. In the Central region mangroves, there was an average of 43.91 ± 39.03 NP/50 cm^2^ and 36.73 ± 56.17 g/50 cm^2^, and for sandy beaches 24.56 ± 35.31 NP/50 cm^2^ and 52.52 ± 56.17 g/50 cm^2^, respectively (more details Table S3). The mean number of plastic items and the mean weight were higher in the Central region for both mangroves and sandy beaches compared to the Western region (Kruskal–Wallis test, *χ*2 = 4.86, df = 1, *p* = 0.02 and *χ*2 = 35.55, df = 1, *p* < 0.01, respectively).

The Kruskal–Wallis statistical test showed differences related to the number of plastic items and weight of plastic litter between the four sites in both regions (NP: *χ*2 = 39.37, df = 3, *p* < 0.01 and weight: *χ*2 = 16.56, df = 3, *p* < 0.01). We found that the Kakun site had the higher number of plastics (935 items) and weight (1029.3 g) recorded, whilst Ankobra had the lowest number (99 items) and weight (442.5 g). Related to the plastic litter between mangrove and sandy beaches, the mangrove regions had an average and standard deviation of 23.60 ± 35.09 NP/50 cm^2^ and 23.65 ± 35.84 g/50 cm^2^, while sandy beaches had 15.87 ± 27.51 NP/50 cm^2^ and 41.52 ± 51.61 g/50 cm^2^. Significant statistical differences were observed in NP (Kruskal–Wallis test, *χ*2 = 11.08, df = 1, *p* < 0.01) and weight of plastic litter (Kruskal–Wallis test, *χ*2 = 6.40, df = 1, *p* = 0.01) between mangroves and sandy beaches.

**Fig. 3:**
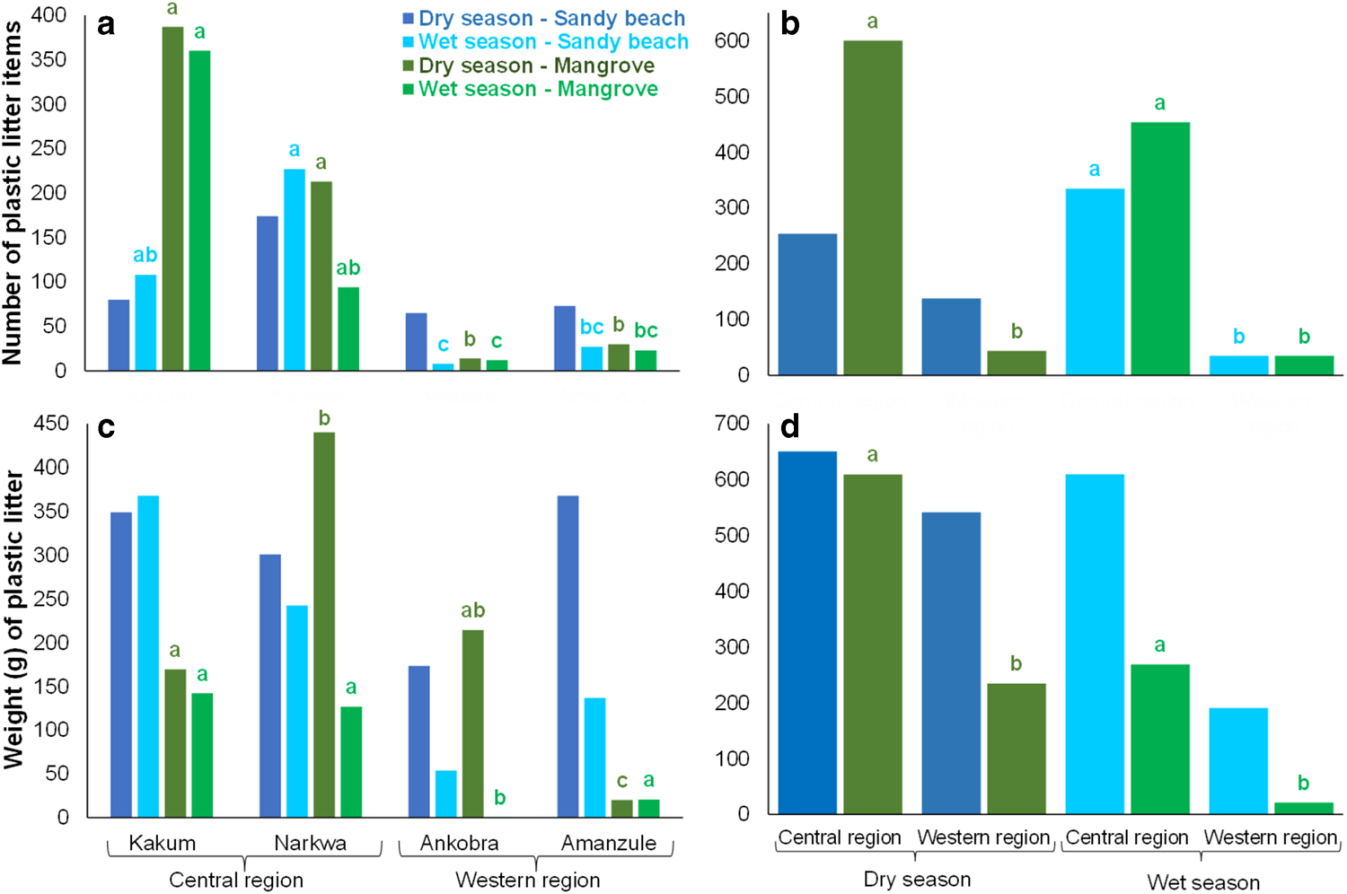
Total number of plastic (NP) litter sampled from 96 quadrants of 50 cm^2^ in the mangrove and sandy beach regions on Central region (Kakum and Narkwa) and Western region (Ankobra and Amanzule) in Ghana, Africa, during dry and wet season periods. **a** Represent the total number of plastics (NP), for the mangrove and sandy beaches in the four sites studied during the wet and dry season. **b** Total number of plastics (NP) for Mangrove and Sandy beaches during the dry and wet periods for the Central and Western region of Ghana. **c** Total weight (g) of the plastic litter for the mangrove and sandy beaches in the four sites studied during the wet and dry season. **d** Total weight (g) of the plastic litter for Mangrove and Sandy beaches during the dry and wet periods for the Central and Western region of Ghana. Differences in NP and weight between the sites (Kakum, Narkawa, Ankobra and Amanzule) and the regions (Central and Western) for wet and dry season are indicated by different letters in same colour (Kruskal–Wallis test, post hoc pairwise Wilcoxon test, *p* < 0.05), see Table 1

Looking at the total NP litter items found during the dry and wet seasons, the dry period had slightly more litter items,1036 plastic items which weighed 2037 g. However, the wet season period had a total of 859 plastic items weighing a total of 1092 g. There was no significant statistical difference for NP litter (Kruskal–Wallis test, *χ*2 = 0.67, df = 1, *p* = 0.41) and weight (Kruskal–Wallis test, *χ*2 = 3.04, df = 1, *p* = 0.08) during the dry and wet period. However, when sandy beaches and mangroves were tested separately to see if there were differences between the dry and wet season period, only the sandy beach regions had a significant statistical difference for the weight of the plastic litter between the seasons (Kruskal–Wallis test, *χ*2 = 7.48, df = 1, *p* < 0.01). The Central region during the dry season had a significant difference for the NP litter items between the mangrove and sandy beaches (Kruskal–Wallis test, *χ*2 = 10.96, df = 1, *p* < 0.01). However, when NP and weight of plastic litter in the mangrove and sandy beaches were tested separately for each site and each region during the dry and wet period, statistical differences were observed (Table 1, Fig. 3). During the dry season period the Central region had a higher abundance of plastic items in the mangroves (600 NP) compared to the sandy beaches (254 NP) (Kruskal–Wallis test,* χ*2 = 10.96, df = 1, *p* < 0.01).

**Table 1:** Kruskal–Wallis test for the number of plastic (NP) litter and weight for sandy beach and mangroves during the dry and wet season period between the four sites studied (Kakum, Narkwa, Ankobra and Amanzule) and between the Central and Western regions of Ghana

NP and weight (g) during the dry and wet season period for sandy beach and mangroves between four sites			
Ecosystems	Season	NP litter items (Fig. 3a)	Weight per grams (Fig. 3c)
Sandy beach	Dry	*χ*2 = 6.44, df = 3, *p* > 0.05	*χ*2 = 2.97, df = 3, *p* > 0.05
Sandy beach	Wet	*χ*2 = 15.95, df = 3, *p* < 0.01***	*χ*2 = 6.79, df = 3, *p* > 0.05
Mangrove	Dry	*χ*2 = 15.19, df = 3, *p* < 0.01***	*χ*2 = 20.62, df = 3, *p* < 0.01***
Mangrove	Wet	*χ*2 = 18.56, df = 3, *p* < 0.01***	*χ*2 = 21.19, df = 3, *p* < 0.01***
NP and weight (g) during the dry and wet season period for sandy beach and mangroves between the Central and Western regions			
Ecosystems	Season	NP litter items (Fig. 3b)	Weight per grams (Fig. 3d)
Sandy beach	Dry	*χ*2 = 0.88, df = 1, *p* > 0.05	*χ*2 = 1.64, df = 1, *p* > 0.05
Sandy beach	Wet	*χ*2 = 13.22, df = 1, *p* < 0.01***	*χ*2 = 2.99, df = 1, *p* > 0.05
Mangrove	Dry	*χ*2 = 13.68, df = 1, *p* < 0.01***	*χ*2 = 5.58, df = 1, *p* = 0.01**
Mangrove	Wet	*χ*2 = 15.35, df = 1, *p* < 0.01***	*χ*2 = 15.79, df = 1, *p* < 0.01***

Significant differences are given by asterisk at 0.05: *p < 0.05; **p < 0.01 and ***p < 0.001 using the Kruskal–Wallis test

### Plastic litter categorisation

**Fig. 4:**
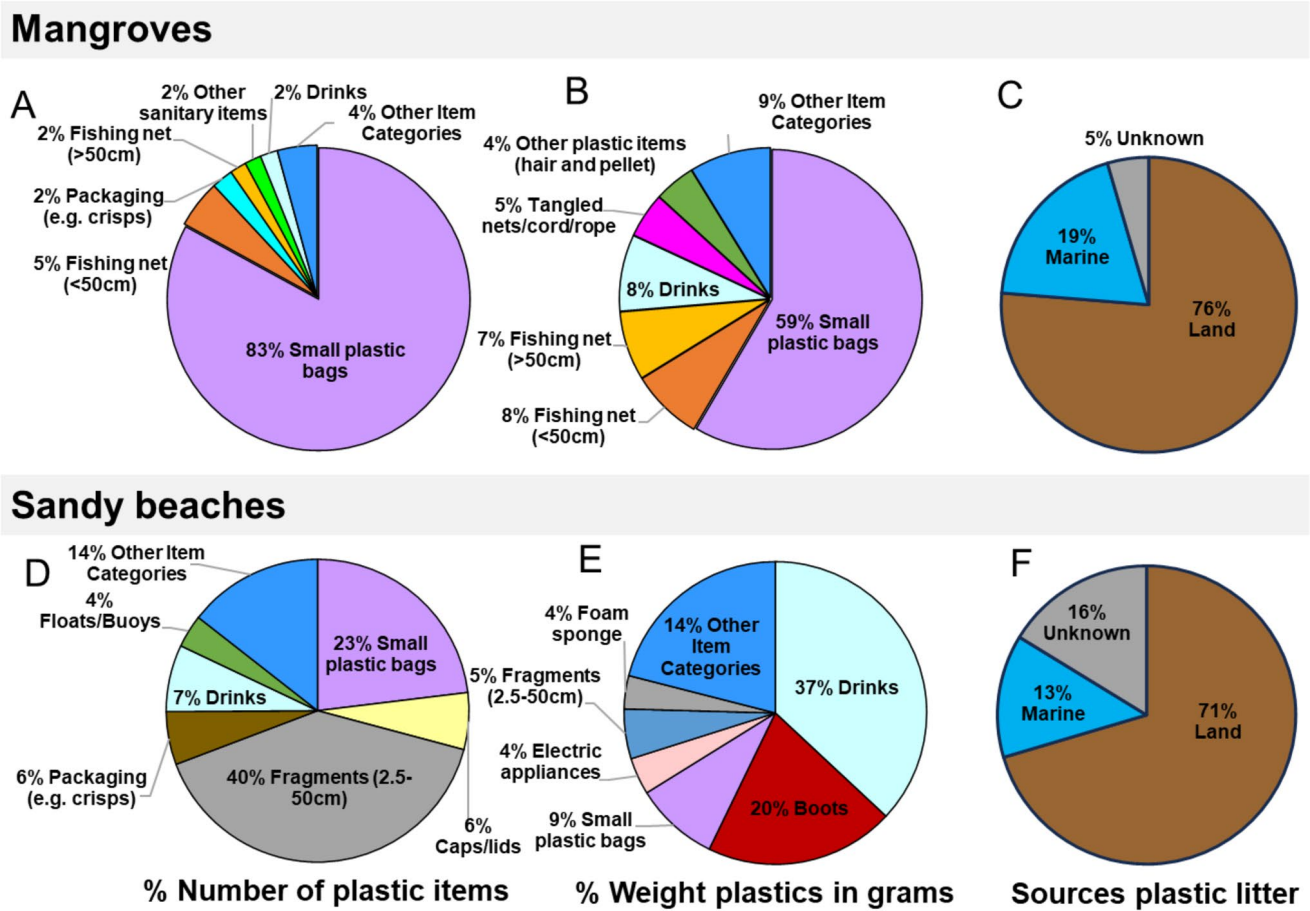
Pie charts showing the percentage of the top six plastic litter categories (displayed), for mangrove and sandy beach regions of Ghana. A/D represents the number of plastic items (NP), B/E represents the weight in grams of the plastic litter items and C/F represents the plastic litter sources type (i.e., land-sourced, marine-sourced, unknown-sourced)

Of the 77 OSPAR plastic litter categories, 30 were found in the study areas, with 26 categories found in the sandy beach regions and 17 in the mangroves. However, some plastic items like water sachets, synthetic hair and nurdles did not have a specific OSPAR category. As such, they were included in the most appropriate category: 4—drinks (bottles, containers and drums) for water sachets, and category 48—other plastic items (synthetic hair and nurdles). The most abundant categories of plastic litter found in our study are: 3/Small plastic bags—59% (mostly represented by plastic films from bags), 46/Plastic/polystyrene pieces 2.5 cm–50 cm—16% (most fragments of polystyrene fast-food containers and hard plastic fragments), 4/Bottles/containers: drinks—4% (including the water sachets) and 115/Nets and pieces of net < 50 cm—4% (Fig. 4, ST1). The heaviest plastics were 3/Small plastic bags (842.97 g), 4/Drinks (bottles, containers and drums (733.79 g)) and 50/Boots (407 g) (Fig. 4, ST1).

### Plastic litter polymers

The plastic polymer for the items that are in the six most abundant categories (Fig. 4) in terms of NP found on the sandy beaches and in the mangroves, and other Plastic Items—hair and other sanitary items—diapers, had the plastic polymer identified, as shown in Table 2. Polyethylene and polypropylene are the most common types of polymers found in our research. Water sachets are composed of PP, and the synthetic hair found is composed of PET. The spectra of the polymers analysed are presented in the Supplementary material—Figure S1.

**Table 2:** Plastic polymers are from the six most abundant plastic litter categories by NP and the categories of other plastic items, such as hair and other sanitary items, such as diapers for the mangrove and sandy beach regions of Ghana

OSPAR category	OSPAR ID	PE	PET	CPE	EVA	PP	PS
Caps/lids	3	3					
Crisp/sweet packets and lolly sticks	4	4	1				
Drinks (bottles, containers, and drums)	15	4	3				
Floats/buoys	19	1			1		2
Fragments (2.5–50 cm)	37			1		4	
Fragments (PS 2.5–50 cm)	46						3
Nets and pieces of net < 50 cm	46	5				2	
Small plastic bags, e.g., freezer bags	115	9					
Other plastic items (hair)	48					1	
Other sanitary items (diaper)	102			1		3	
**Grand total**		**26**	**4**	**2**	**1**	**10**	**5**

*PE* polyethylene + polyethylene isotactic, *EVA* ethylene–vinyl acetate, *PET* polyethylene terephthalate, *CPE* polyethylene chlorinated, *PP* polypropylene + polypropylene isotactic, *PS* polystyrene

### Input source characterisation

The sources of the plastic litter from all regions collected at sandy beaches and mangrove sites in our data have been identified following the survey protocol of the Marine Conservation Society and OSPAR guidelines (OSPAR Commission 2020; Allison et al. 2023). Our results indicate that terrestrial input is responsible for more than 70% of the plastic litter found in the mangroves and coats, followed by marine sources > 13% (Fig. 4).

## Discussion

The results presented in our study show the pervasive presence of plastics in Ghanaian mangroves and sandy beaches, with mangroves having a higher number of plastics, while the sandy beaches had plastics that generally weighed more. In total, our study found, on average, 94 NP/m^2^ in the mangroves, whilst sandy beaches were slightly lower with 63 NP/m^2^. This study shows that the most urbanised area, the Central region of Ghana, has the highest abundance of plastic litter. Our results also indicated seasonal variation in number and weight of plastic litter, between the mangroves and sandy beaches depending on the site/region studied. Plastic bags, bottle drink containers, plastic fragments and nets were among the most frequent items found in our study and were mainly composed of polyethylene and polypropylene polymers. The sources of plastic litter are primarily land-based. Based on the OSPAR guide (Wenneker and Oosterbaan 2010), the plastic litter found in our study represents around 40% of the plastic waste categories classified in the guide. However, the guide does not have a specific classification for plastic litter items which are representative of Ghana and that characterises some specific uses of plastics in the country.

Our results highlight that the concentration of plastic litter on Ghanaian sandy beaches in the Western (averaging from 6 to 35 NP/m^2^) and Central regions (averaging from 52 to 94 NP/m^2^) was higher than the global sandy beach debris average of 1 item/m^2^ (Galgani et al. 2015). According to Gbogbo et al. (2023), the deposition rate of waste plastic on Ghana’s sandy beaches is 8.1 ± 2.5 plastic items per m^2^, with a weight of 0.35 ± 0.11 kg plastics per m^2^. Most of the plastic debris and particles are washed into the intertidal zones of the sandy beach, which occurs during the highest tide, with the litter remaining behind when the tide recedes (Zahari et al. 2022). The number of plastic litters in our study come from the surface and top 2.5 cm of the sediment; this could increase with greater sediment depth. Tavares et al. (2020) demonstrated that plastic debris buried at 10 cm was 25 times higher (48.75 items/m^2^) than that observed at the surface (1.92 items/m^2^) in Senegal, West Africa. In our study, Elmina Castle (Kakum site) and the Ankobra Beach Resort (Ankobra site), both of which are known tourist areas, had undergone beach clean-up efforts, which are undertaken daily each morning using a lawn rake. This method collects big pieces of plastic litter (> 3 cm) from just on/in the surface of the sandy. However, the number of plastic litters in these areas did not differ significantly from the other sampled points without cleaning efforts. This may be due to the insufficient effectiveness of the cleaning efforts, as indicated by Gbogbo et al. (2023), regarding plastic litter deposition in each tidal cycle at the beaches.

Mangroves in our study had the highest number of plastics trapped compared to the sandy beach. Regarding plastic litter concentration in the Ghanaian mangroves, only some regions in Indonesia as Kendari Bay ~ 252 NP/m^2^ (Rahim et al. 2020) and Arbon ~ 92 NP/m^2^ (Suyadi and Manullang 2020) had higher or similar concentrations of plastics when compared to our study (94 NP/m^2^). In our study, the depth of sampling and the inclusion of fragments greater than 0.5 cm in size could be factors that increased the number of plastic items found in the Ghanaian mangroves compared to other studies. However, undertaking direct comparisons of plastic litter abundance between different regions and countries can be problematic as there are many other reasons that can influence quantities of plastic litter, including location, in-country waste management schemes, the culture of individual countries and communities, how plastic accumulates over time in different locations, sampling collections, etc.

The higher accumulation of plastic litter in the mangroves could be related to the tree morphology, the tree’s roots and trunks, and pneumatophores trap higher quantities of plastics (Cappa et al. 2023). It has been described to be responsible for most plastic retention in this ecosystem (Martin et al. 2019; 2020; Walther and Bergmann 2022; Cappa et al. 2023). Most of these plastics become buried over time by the high levels of organic matter deposition, affecting the ecosystem’s functioning (Martin et al. 2019; 2020; Walther and Bergmann 2022). Plastic pollution at the mangroves can cause entanglement with the vegetation. It impairs plant respiration and photosynthesis, damages seedlings and leads to seedling mortality and potential tree death (Suyadi and Manullang 2020). The mangroves in our study were found to trap huge amounts of plastic bags which become easily tangled in the trees. Mangrove morphology makes litter cleaning programs in these areas difficult; preventing waste from entering this ecosystem in the first instance would be the most effective strategy (Suyadi and Manullang 2020).

In our study, the urbanisation level and population density, as well as general preservation in the regions, are likely factors that directly influence the abundance of plastic litter in the studied regions. There was a reduced number of plastic litter items in the Western region than in the Central region. The Western region in Ghana is strongly influenced by cultural beliefs that impact people’s behaviour to preserve and respect nature. One of these symbols to the Ghanaian people is called “Sankofa”—“return to the root” (Tettey 2018), which is a belief that they need to respect all that god has created (Essien 2003). The Sankofa symbol educates society members to follow the paths of their ancestors by caring for the environment and engaging in healthy environmental practices (Adom et al. 2018). We believe that this is one of the cultural influences that have most likely led to lower levels of plastic litter on the Western sites in Ghana—in addition to lower population density in this region. According to Suyadi and Manullang (2020), levels of plastic waste are related to the distance from city centres, industries and markets and have also been associated with the human population around mangroves and sandy beach regions. However, other factors such as maritime activity, surface currents, tides, watershed outfalls and wind affect levels of plastic pollution (Eriksen et al. 2014).

Dry and wet season period showed differences on the number and weight of plastic litter for the mangroves and sandy beaches when tested separated between the sites and regions. At the Central region this study found a higher abundance of plastic items in the mangroves compared to the sandy beaches during the dry season. Nonetheless, this tends to reverse during the wet season, when plastic litter increased in wet season on the sandy beaches relative to the dry season, as found by Adu-Boahen (2024) in Abandze, Anomabo and Biriwa beaches in the Central region of Ghana. This is probably related to the waste washed from land to the sea, especially in the wet season; according to the Ghana National Plastic Action Partnership (NPAP) (2021), ~ 50% of the plastic litter is directly dumped on land.

The plastic litter input into the oceans comes mostly from primary routes such as rivers and mangroves (Meijer et al. 2021). Haberstroh et al. (2021) studied the plastic transport mechanisms on river flows and found that plastic transport was governed by bulk motion advection; low-density polymers tended to remain at the surface. The impact of turbulence on plastic particles depends on properties such as size, shape and composition. de Almeida Duarte et al. (2023), studying the waste transport on Santos Channel in Brazil, found that hydrodynamic conditions revealed that tidal currents transport waste from the interior of the mangrove forests to the beaches, therefore indicating that the mangroves themselves act as a marine litter hotspot.

Tide and water currents in the landward contribute to plastic retention in the beach and mangrove zones (do Sul et al. 2014; Gbogbo et al. 2023). The Ghanaian coastal environment experiences an annual average high tide of 1.70 m and a low tide of 0.17 m (Dei 2010), which can cover greater distances on land due to the gently sloping nature of the coastline and, as a result, transport plastics further on to the beach. The plastic litter transport and accumulation processes are influenced by plastic and environmental characteristics (Haberstroh et al. 2021; de Almeida Duarte et al. 2023). Ghanaian coastal waters are influenced by the Guinea Current that extends from Bissagos Island (Guinea Bissau) in the north to Cape Lopez (Gabon) in the south. The Guinea Current is primarily driven by winds, particularly the southwest trade winds, which push surface waters along the West African coast (Ukwe et al. 2006; Neokye et al. 2021). This current can carry floating plastic debris from the open ocean towards the coastal regions. The seasonal movement of the Intertropical Convergence Zone (ITCZ) affects the strength and position of the Guinea Current. The ITCZ is a planetary-scale band of rising air and intense rainfall near the equator that migrates seasonally (Mamalakis et al. 2021). During the rainy season, the ITCZ shifts northward (Nicholson 2009), leading to stronger trade winds and a more pronounced Guinea Current. This strengthened current can enhance the transport of plastics along the coast of West Africa, dispersing debris more effectively. Conversely, in the dry season, the ITCZ moves southward (Nicholson 2009), weakening the current and potentially reducing the efficiency of plastic transport. In this way, during the wet season (summer), the Ghanaian coast would be expected to have higher quantities of debris accumulating according to the current strength compared to the dry season (winter). However, Guinea’s current adds to the ITCZ influence on plastic litter transport and distribution needs more studies to help explain the litter pollution on the West coast of Africa, as well as in Ghana.

Ghana’s current efforts to address plastic pollution are insufficient (Ghana National Plastic Action Partnership (NPAP), 2021). The most common methods of plastic waste disposal are open dumping, followed by landfilling and burning (Ghana Statistical Service 2021). The most prevalent plastic litter input in our study originated from land sources (Awewomom et al. 2024). Similar results were found for beaches along Ghana’s Accra-Tema sandy beachline by Van Dyck et al. (2016), where 93% of all debris collected was found to have originated from land, with the remaining 1% from the ocean and 6% from unknown sources. Zielinski et al. (2022) found that globally, the most common litter comes from land sources; the global ratio for land-based and sea-based sources of marine litter is 80% and 20%, respectively.

Our analysis revealed that plastic bags, drink containers, fishing materials and plastic fragments were the most abundant plastic items found. The results showed that low-density plastics such as plastic bags were more likely to become trapped by the tree structures and buried in the mangroves (do Sul et al. 2014; Martin et al. 2019), while on the sandy beaches, plastic bottles and fragments were prevalent but covered different polymer types from low-density polystyrene and polypropylene through to higher-density polyethylene terephthalate. However, it was possible to notice that most plastics found on the beaches could fluctuate according to their density or because of the air trapped inside. do Sul et al. (2014) rarely found plastic bottles floating away from the mangroves on the tide, while bags were found entangled by the roots. Van Dyck et al. (2016) found that plastic bottles, black plastic bags, pure water sachets and food wrappers were the dominant litter types found on Ghanaian sandy beaches, which is similar to our results. Many West African countries use drinkable water sold in plastic sachets and bottles (single-use plastics), generating indiscriminate plastic litter disposal everywhere (Akindele and Alimba 2021). In Ghana, plastic bag waste is the most common plastic waste generated by households in Accra (Awewomom et al. 2024), followed by plastic bottles/water sachets and food packaging materials (Jonathan et al. 2023). The West Africa market also contributes with huge amounts of plastic bags litter (Akindele and Alimba 2021). Small plastic bags are one of the most representative plastic litter items in our study, being the most abundant item in the mangroves and one of the six most abundant found on sandy beaches. Similar results were also observed on Indian Mumbai mangroves (Kesavan et al. 2021), Southeast sandy beaches (Jeyasanta et al. 2020), sandy beaches in the Santos estuary in Brazil (Cordeiro and Costa 2010) and for Cebu Island in the Central Philippines (Paler et al. 2022). The other two items that were representative in our study were nets, mostly in the mangrove, and fragments (2.5–50 cm), most on the sandy beach. Our results corroborate those of Kesavan et al. (2021) who also found nets and fishing gear in the mangroves, allowing more debris to be trapped inside it, increasing plastic pollution. Jeyasanta et al. (2020) also found high numbers of polystyrene foam fragments and food containers on Indian beaches.

Polyethylene and polypropylene in our study are the most representative plastic polymers found in bags, lids, food wrapping, drink containers, nets, plastic fragments, diapers and synthetic hair. These polymers are widely used plastic materials, contributing to nearly 60% of the world’s plastic production (~ 400 million tonnes), most single-use plastic (Geyer et al. 2017). The recycling of these plastic polymers is possible but could have at least twice the market value of virgin plastics, making it infeasible for companies to reuse them (Xue et al. 2021). Hoseini and Bond (2022) estimated that 3.2 ± 1.8 Mt/year of polyethene, 1.3 ± 0.8 Mt/year of polypropylene and 1.6 ± 0.9 Mt/year of polyethene terephthalate enter the environment annually. The prediction of the most abundant plastic polymers released in the environment comprises 20% of PE, 14% of PET and 11% of PP of the total accumulated plastic (Hoseini and Bond 2022).

Using the OSPAR guide (Wenneker and Oosterbaan 2010) to categorise the plastic items found in the Ghanaian mangroves and sandy beaches did not allow us to place some of our plastic items into the adequate existing categories, as no suitable category accurately reflected the plastic waste found in Ghana. Consequently, not all plastic litter items were not properly categorised, which may have resulted in under/over-estimation of the true scale of plastic pollution sources in Ghana. The absence of litter categories pertinent to a specific country or region of the world in the OSPAR guide will influence the overall picture regarding litter types in the country. This study shows that water sachets and synthetic hair could not be allocated to an appropriate OSPAR category. These plastic items are key indicators of Ghanaian development and culture. The consumption of water from sachets is prevalent in Ghana due to their affordability and convenience for accessing clean drinking water (Moulds et al. 2022). Similarly, hair extensions and braids, commonly worn by many women in Western Africa, are often made from synthetic polymers (Evans-Anfom 2014). Synthetic hair extensions are commonly changed every month (Thomas 2023), generating increased levels of plastic fibres entering into the environment. Gonçalves et al. (2025) found that high levels of microplastic fibres and fragments are probably as a result of synthetic hair and water sachets/plastic bags respectively in the mangrove environment. To date little attention has been given to synthetic hair as a source of plastic pollution entering the environment, and as such little is known regarding the potential impact on the ecosystem as well as human health. Based on our results, we suggest that the OSPAR guide, which is based on litter items collected from the North-East Atlantic, should consider including categories that are relevant to other regions of the world. This would better represent the main sources of plastic pollution in different countries and regions.

This study adapts and uses simple and well-recognised survey methods such as quadrats and the OSPAR guide, to estimate plastic litter in mangroves and sandy beaches in Ghana. Both methods were used together to enhance the survey’s comprehensiveness and the accuracy of the results. However, applying the OSPAR beach cleaning methodology in mangrove regions poses challenges due to the barriers created by trees, as well as the organic matter deposition that covers the plastics, leading to an underestimation of plastic litter. Lenaker et al. (2019) highlight that focusing just on surface sampling may poorly estimate the true magnitude of plastic loads in water or buried in the sediment of mangroves. For this reason, we sampled the top 2.5 cm of sediment in the quadrats surveyed. We are confident that these methods effectively demonstrated the levels of plastic litter in mangroves and sandy beach regions in this study.

## Conclusion

The study highlights the widespread presence of plastic litter in Ghana’s mangrove and sandy beach areas. The Central region of Ghana presented a higher abundance of plastic litter, which can be attributed to urbanisation and higher population density which leads to a higher concentration of plastic litter, in contrast to the Western region. Additionally, our study also shows that seasonal variations influence plastic litter accumulation, between mangroves and sandy beach according to the sites and regions. The plastic litter found in Ghana had less diversity of plastic litter categories when compared to OSPAR guide categories. However, Ghana had some characteristics of plastic litter that were not categorised in OSPAR, which could have underestimated and hidden important results. Notably, land-based sources such as plastic bags, bottle drink containers and plastic fragments (mostly made of polyethylene and polypropylene polymers) were the most prevalent items found in Ghana’s coastal areas. These insights underscore the urgent need for targeted interventions to mitigate plastic pollution in Ghana’s coastal ecosystems. The significant plastic pollution in Ghana is linked to poor waste management, a lack of clean drinking water and high consumption of single-use plastics. These funds are also reflections of the plastic pollution in West Africa due to the lack of effective waste management increasing the waste and worsening marine pollution. Plastic pollution negatively impacts the fishery community in Ghana and West Africa in general, by reducing food and income for these communities. It also causes mangrove degradation, affecting mangrove trees’ generation and survival. In mangroves and sandy beach areas, plastic affects the fauna associated with the ecosystems as well by reducing the foraging area, causing entanglement and/or ingestion of plastics. The Ghanaian government and West African leaders must take urgent action to preserve its rich coastal ecosystems. These include implementing clean sources of piped water, improving waste management, and decreasing or banning single-use plastics. Such measures are essential for ecosystem preservation, environmental regeneration and sustaining community benefits. Further research and conservation efforts should focus on comprehensively assessing the impacts of plastic pollution on the sandy beach and mangrove species and ecosystems, developing effective mitigation strategies, and raising awareness about the importance of preserving these unique and vital environments in Ghana and West Africa.

## Supplementary Information

Below is the link to the electronic supplementary material.

Supplementary file1 (DOCX 372 KB)

## Data Availability

Data will be made available on request.
