# Supplementary material for: Patterns and implications of plastic accumulation in mangrove ecosystems and sandy beaches in Western and Central regions of Ghana, West Africa
Source: Environ Sci Pollut Res Int. 2025 Apr 22;32(19):11996–2012. doi: 10.1007/s11356-025-36359-7 (PMC12049385; doi:10.1007/s11356-025-36359-7)
Supplement: Supplementary file 1 — Supplementary file1 (DOCX 372 KB) [file 11356_2025_36359_MOESM1_ESM.docx]

**SUPPLEMENTARY MATERIAL**

1. **Supplementary Tables**

**Table S1:** Detailed description of the division of plastic litter categories into source type and sub-source, as well as the percentage each represents across the mangrove and sandy beaches regions in Ghana - Africa. A number of plastics (NP) and average weight (g), including minimum and maximum values, have been calculated for the plastic litter category.

| **Source**  **Type** | **Sub-Source** | **Item** | **OSPAR ID** | **OCPP ID** | **NP** | **% Count** | **% Weight** | **Total**  **Weight (g)** | **Av. Weight**  **(g) / Item** | **Min (g)** | **Max (g)** |
| --- | --- | --- | --- | --- | --- | --- | --- | --- | --- | --- | --- |
| **Land** | **Public** | Bags (shopping) | 2 | 2 | 1 | 0.05 | 0.22 | 7 | 7 | 0.00 | 7 |
|  |  | Small plastic bags | 3 | D01 | 1111 | 58.63 | 26.98 | 843.97 | 0.76 | 0.00 | 114 |
|  |  | Water sachet | NA-4 | 4 | 38 | 2.01 | 3.29 | 102.92 | 2.71 | 0.13 | 37 |
|  |  | Bottles / containers: drinks | 4 | 4 | 36 | 1.90 | 23.45 | 733.79 | 20.38 | 1.60 | 111.89 |
|  |  | Bottles / containers: toiletries | 7 | 7 | 9 | 0.47 | 1.41 | 44.20 | 4.91 | 0.15 | 13.12 |
|  |  | Caps / lids | 15 | 15 | 26 | 1.37 | 1.85 | 57.88 | 2.23 | 10.94 | 26.14 |
|  |  | Cigarette lighters / tobacco pouches | 16 | 16 | 1 | 0.05 | 0.35 | 10.94 | 10.94 | 0.00 | 10.94 |
|  |  | Packets (crisps / lolly / sweets) | 19 | G01 | 68 | 3.59 | 0.97 | 30.38 | 0.45 | 2.27 | 10.02 |
|  |  | Cups (plastic) | 21 | A01 | 6 | 0.32 | 0.58 | 18.02 | 3 | 0.38 | 5.44 |
|  |  | Cutlery / tray / straw | 22 | 22 | 2 | 0.11 | 0.03 | 1.09 | 0.55 | 0.75 | 0.71 |
|  |  | Tablecloth plastic sheeting | 40 | 40 | 9 | 0.47 | 0.02 | 0.75 | 0.08 | 9.61 | 0.75 |
|  |  | Shoes/sandals | 44 | L01 | 1 | 0.05 | 0.31 | 9.61 | 9.61 | 28.31 | 9.61 |
|  |  | Boots | 50 | L01 | 4 | 0.21 | 13.01 | 407.00 | 101.75 | 0.37 | 205.8 |
|  |  | Other rubber | 53 | P01 | 7 | 0.37 | 0.45 | 14.17 | 2.02 | 0.00 | 6.37 |
|  |  | Clothing | 54 | 54 | 19 | 1.00 | 2.16 | 67.63 | 3.56 | 79.38 | 32.4 |
|  |  | Phone charger | 79 | 79 | 2 | 0.11 | 2.54 | 79.38 | 39.69 | 0.01 | 79.38 |
|  | **Sewage** | Diaper | 102 | X01 | 20 | 1.06 | 0.85 | 26.66 | 1.33 | 17.36 | 17.2 |
| **Marine** | **Fishing** | String / cord / rope (thickness >1 cm) | 31 | 31 | 4 | 0.21 | 1.22 | 38.25 | 9.56 | 0.09 | 20.89 |
|  |  | String / cord / rope (thickness 0 – 1 cm) | 32 | I01 | 9 | 0.47 | 0.59 | 18.46 | 2.05 | 0.04 | 8.6 |
|  |  | Tangled nets / cord / rope / string / dolly rope | 33 | 331 | 13 | 0.69 | 3.28 | 102.69 | 7.90 | 0.01 | 50 |
|  |  | Floats / buoys | 37 | 37 | 26 | 1.37 | 1.32 | 41.15 | 1.58 | 0.00 | 13.11 |
|  |  | Fishing net (0-50 cm) | 115 | J01 | 77 | 4.06 | 3.04 | 95.09 | 1.23 | 43.40 | 49.46 |
| **Unknown** | **Non-Sourced** | Bottles / containers: drums | 12 | 12 | 1 | 0.05 | 1.39 | 43.40 | 43.40 | 0.03 | 43.4 |
|  |  | Foam / sponge / insulation | 45 | 45 | 7 | 0.37 | 2.29 | 71.59 | 10.23 | 0.07 | 71 |
|  |  | Synthetic hair | NA - 48 | O01 | 4 | 0.21 | 1.61 | 50.27 | 12.57 | 0.09 | 12.22 |
|  |  | Pellets (nudles) | NA - 48 | O01 | 25 | 1.32 | 0.03 | 0.83 | 0.03 | 0.00 | 0.43 |
|  | **Fragments** | Fragments (ply 2.5 – 50 cm) | 46 | N01 | 191 | 10.08 | 1.07 | 33.34 | 0.17 | 0.06 | 13.2 |
|  |  | Fragments (plastic 2.5 – 50 cm) | 46 | M01 | 111 | 5.86 | 2.46 | 76.93 | 0.69 | 0.67 | 19.44 |
|  |  | Fragments (ply >50 cm) | 47 | N01 | 21 | 1.11 | 0.45 | 13.98 | 0.67 | 0.02 | 10.14 |
|  |  | Nets and pieces of net > 50 cm | 116 | J01 | 20 | 1.06 | 2.70 | 84.48 | 4.22 | 0.00 | 35.25 |
|  |  | Fragments (plastic 0 – 2.5 cm) | 117 | M01 | 26 | 1.37 | 0.09 | 2.81 | 0.11 | 0.00 | 0.66 |
|  |  |  |  |  |  |  |  |  |  |  |  |

**Table S2.** Kruskal-Wallis test presents the absence of difference related to the number of plastic items and plastic weight between the sampling points (right and left of the river connection to the sea) in the sandy beaches of Kakum and Ankobra.

| Site | Variable | χ2 | df | P |
| --- | --- | --- | --- | --- |
| Kakum | Number of plastic | 0.12 | 1 | 0.72 |
| Kakum | Weight | 0.008 | 1 | 0.97 |
| Ankobra | Number of plastic | 0.76 | 1 | 0.38 |
| Ankobra | Weight | 1.35 | 1 | 0.24 |

**Table S3.** Average number of plastics (NP), weight (g) and standard deviation (SD) per 50cm^2^ in mangrove and sandy beaches surveyed in Westen and Central regions.

|  |  | Mangrove | | Sandy beach | |
| --- | --- | --- | --- | --- | --- |
| Regions | Sites | NP/50cm^2^ | Weight g/50cm^2^ | NP/50cm^2^ | Weight g/50cm^2^ |
| Central region | Kakum | 31.13 ± 40.82 | 7.83 ± 18.28 | 13.01 ± 15.52 | 29.87 ± 18.28 |
|  | Narkwa | 12.79 ± 26.61 | 16.70 ± 45.77 | 23.62 ± 45.83 | 22.64 ± 45.77 |
| West region | Ankobra | 1.08 ± 1.63 | 3.04 ± 44.10 | 8.96 ± 7.95 | 9.47 ± 44.10 |
|  | Amanzule | 2.20 ± 3.61 | 4.16 ± 4.96 | 1.70 ± 6.63 | 21.05 ± 4.96 |

**Figure S1.** Plastic polymer spectra from the 6 most abundant plastic litter items, and synthetic hair and other sanitary items - diapers, that are found on the sandy beaches and mangroves on the Ghanaian coast.
